# Nephrotoxic Potential of Putative 3,5-Dichloroaniline (3,5-DCA) Metabolites and Biotransformation of 3,5-DCA in Isolated Kidney Cells from Fischer 344 Rats

**DOI:** 10.3390/ijms22010292

**Published:** 2020-12-30

**Authors:** Gary O. Rankin, Christopher R. Racine, Monica A. Valentovic, Dianne K. Anestis

**Affiliations:** Department of Biomedical Sciences, Joan C. Edwards School of Medicine, Marshall University, Huntington, WV 25755, USA; racine.christopher@yahoo.com (C.R.R.); valentov@marshall.edu (M.A.V.); anestis@marshall.edu (D.K.A.)

**Keywords:** nephrotoxicity, in vitro, rat, 3,5-dichloroaniline, 3,5-dichlorophenylhydroxylamine, 3,5-dichloroacetanilide, 2-amino-4,6-dichlorophenol, 3,5-dichloronitrobenzene

## Abstract

The current study was designed to explore the in vitro nephrotoxic potential of four 3,5-dichloroaniline (3,5-DCA) metabolites (3,5-dichloroacetanilide, 3,5-DCAA; 3,5-dichlorophenylhydroxylamine, 3,5-DCPHA; 2-amino-4,6-dichlorophenol, 2-A-4,6-DCP; 3,5-dichloronitrobenzene, 3,5-DCNB) and to determine the renal metabolism of 3,5-DCA in vitro. In cytotoxicity testing, isolated kidney cells (IKC) from male Fischer 344 rats (~4 million/mL, 3 mL) were exposed to a metabolite (0–1.5 mM; up to 90 min) or vehicle. Of these metabolites, 3,5-DCPHA was the most potent nephrotoxicant, with 3,5-DCNB intermediate in nephrotoxic potential. 2-A-4,6-DCP and 3,5-DCAA were not cytotoxic. In separate experiments, 3,5-DCNB cytotoxicity was reduced by pretreating IKC with antioxidants and cytochrome P450, flavin monooxygenase and peroxidase inhibitors, while 3,5-DCPHA cytotoxicity was attenuated by two nucleophilic antioxidants (glutathione and N-acetyl-L-cysteine). Incubation of IKC with 3,5-DCA (0.5–1.0 mM, 90 min) produced only 3,5-DCAA and 3,5-DCNB as detectable metabolites. These data suggest that 3,5-DCNB and 3,5-DCPHA are potential nephrotoxic metabolites and may contribute to 3,5-DCA induced nephrotoxicity in vivo. In addition, the kidney can bioactivate 3,5-DCNB to toxic metabolites, and 3,5-DCPHA appears to generate reactive metabolites to contribute to 3,5-DCA nephrotoxicity. In vitro, N-oxidation of 3,5-DCA appears to be the primary mechanism of bioactivation of 3,5-DCA to nephrotoxic metabolites.

## 1. Introduction

Chloroanilines are chemical intermediates commonly used in the manufacturing process for many dyes, agricultural chemicals, drugs and industrial chemicals. Exposure to chloroanilines occurs in occupational settings, via their release or formation during mammalian metabolism of numerous compounds [1,2,3], or by environmental degradation of pesticides [1,4,5,6]. The detection of chloroanilines in human urine or blood has been used as a biomarker for exposure to chloroaniline-based pesticides [7,8,9,10]. Toxicity associated with exposure to chloroanilines includes hematotoxicity (e.g., anemia or methemoglobinemia) [11,12,13], splenotoxicity [11,14], hepatotoxicity [14,15,16,17], endocrine disruption [18] and nephrotoxicity [16,19,20]. Chloroanilines are also considered priority pollutants in environmental risk assessment studies because of their potential adverse health effects and release following pesticide degradation into the environment in agricultural areas [21,22].

Among the three monochlorinated and six dichlorinated anilines, 3,5-dichloroaniline (3,5-DCA) is the most potent nephrotoxicant in vivo and in vitro [16,19,23]. In vivo exposure to 3,5-DCA in Sprague–Dawley or Fischer 344 rats resulted in an elevated blood urea nitrogen (BUN) concentration, decreased organic ion transport in proximal tubular cells, decreased kidney weight, proteinuria, and hematuria [23,24]. The most severe morphological changes occur in the renal proximal tubular cells, but the distal tubular cells and collecting ducts are also affected by 3,5-DCA exposure [23]. Further studies using isolated kidney cells (IKC) from male Fischer 344 rats demonstrated that concentrations of 1.0 mM or greater 3,5-DCA exposure for 90 min leads to increased lactate dehydrogenase (LDH) release, a measure of cytotoxicity [25].

**Figure 1:**
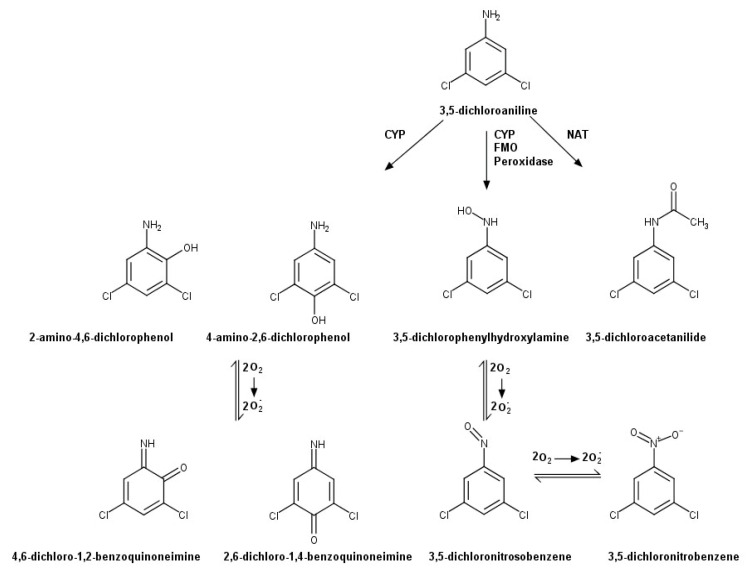
Proposed Biotransformation Pathway of 3,5-DCA. Abbreviations: CYP = Cytochrome P450, FMO = Flavin-containing Monooxygenase, NAT = N-acetyltransferase.

Toxicity of aniline compounds in vivo is mainly due to the formation of toxic metabolites. Based on studies with aniline and some of its chlorinated derivatives, the biotransformation pathways for these aniline compounds have been clearly established and include N-oxidation, N-acetylation, and phenyl ring oxidation [13,26,27,28]. Although the in vivo metabolism of 3,5-DCA has not been studied in any detail, these biotransformation pathways could produce a number of potentially toxic metabolites (Figure 1), as occurs with other chloroanilines where biotransformation studies have been conducted [26,29,30]. Recent studies by Racine et al. [25] have demonstrated that 3,5-DCA is metabolized to toxic metabolites via a number of renal enzyme systems. However, it is not clear which metabolites are produced by kidneys.

One potential 3,5-DCA metabolite, 4-amino-2,6-dichlorophenol (4-A-2,6-DCP), has been evaluated for nephrotoxic potential and is a potent nephrotoxicant in vivo and in vitro in male Fischer 344 rats [31,32,33]. A comparative in vitro study of the nephrotoxic potential of ten mono-, di- and trichloronitrobenzenes in renal cortical slices found 3,5-dichloronitrobenzene (3,5-DCNB) to be the least potent nephrotoxicant among the dichloronitrobenzenes [34]. To date, there are currently no studies that have explored the nephrotoxic potential of other putative 3,5-DCA metabolites.

The current study was designed to determine the nephrotoxic potential of three additional putative 3,5-DCA metabolites that arise from N-acetylation, (3,5-dichloroacetanilide; 3,5-DCAA), N-oxidation (3,5-dichlorophenylhydroxylamine; 3,5-DCPHA), or phenyl ring oxidation (2-amino-4,6-dichlorophenol; 2-A-4,6-DCP) (Figure 1) following extra-renal biotransformation of 3,5-DCA. The nephrotoxic potential of 3,5-DCNB in the IKC model and its potential mechanism(s) of bioactivation were explored. In addition, the potential for free radicals/reactive metabolites to contribute to 3,5-DCPHA cytotoxicity was determined. Lastly, a biotransformation study was conducted to ascertain to what extent 3,5-DCA is biotransformed in IKC from male Fischer 344 rats and to determine which metabolites are formed.

## 2. Results

### 2.1. Time and Concentration Cytotoxicity Relationships for 3,5-DCA Metabolites

**Figure 2:**
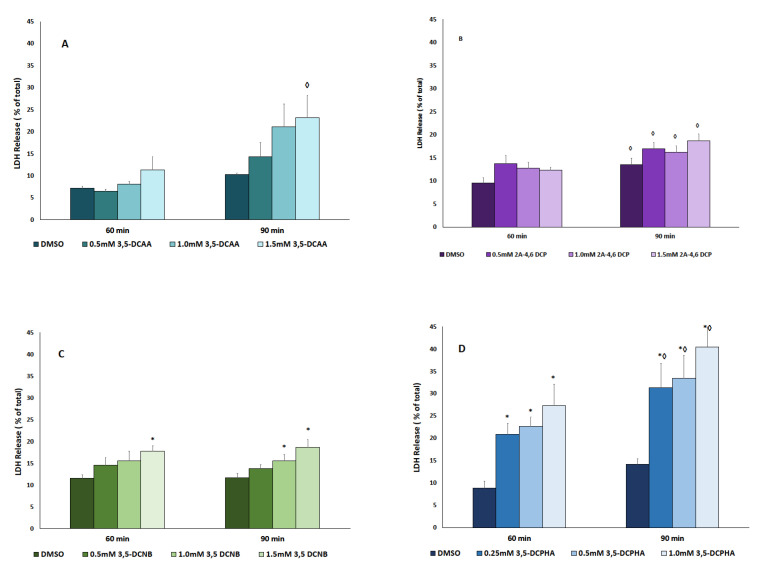
Cytotoxicity induced by 3,5-DCAA (**A**), 2-A-4,6-DCP (**B**), 3,5-DCNB (**C**) or 3,5-DCPHA (**D**) following exposure for 60–90 min. Each bar represents the mean ± S.E.M. for N = 4–5 separate isolation experiments. * Significantly different from DMSO control, *p* < 0.05. ◊ Significantly different from 60 min value, *p* < 0.05.

To determine the nephrotoxic potential of each 3,5-DCA putative metabolite, concentration and time course studies were conducted in IKC. Exposure to 3,5-DCAA did not significantly increase LDH release, a marker of cytotoxicity, at any time (60 or 90 min) or concentration (0–1.5 mM) tested (Figure 2A). In addition, exposure to 2-A-4,6-DCP did not result in significant increases in LDH release at any concentration tested (up to 1.5 mM) after a 60 or 90 min exposure (Figure 2B). Incubation of IKC with 3,5-DCNB or 3,5-DCPHA resulted in time and concentration dependent increases in LDH release for both compounds, although 3,5-DCPHA induced the largest increases in LDH release. Significant LDH release was seen at 3,5-DCNB concentrations 1.0 or 1.5. mM or greater after 90 min but at only 1.5 mM after 60 min (Figure 2C). In contrast, 3,5-DCPHA induced a significant increase in LDH release at 0.25 mM or greater after 60 and 90 min, with the increases in LDH release observed at 90 min being significantly increased as compared to respective 60 min increases in LDH release (Figure 2D). Therefore, of the metabolites tested, 3,5-DCPHA was the most nephrotoxic.

### 2.2. Renal Biotransformation and 3,5-DCNB Cytotoxicity

**Figure 3:**
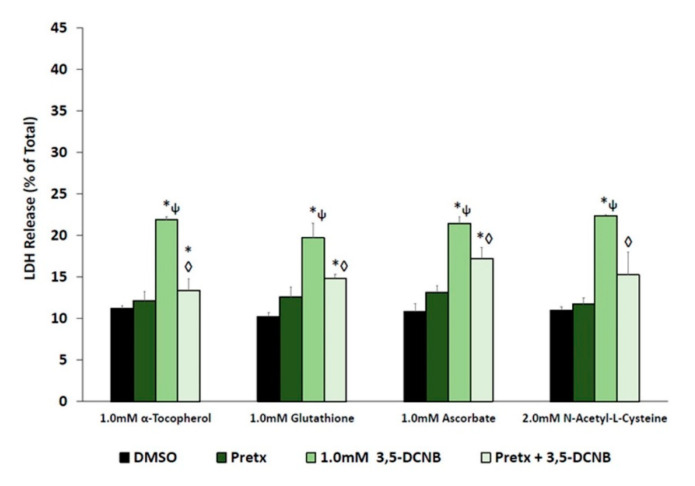
Effects of antioxidant pretreatment (Pretx) on 3,5-DCNB induced cytotoxicity in isolated kidney cells obtained from male Fischer 344 rats after 60 min. Each bar represents the mean ± S.E.M. for N = 4–5 separate isolation experiments. * Significantly different from DMSO control, *p* < 0.05. ◊ Significantly different from the 1.0 mM 3,5-DCNB value, *p* < 0.05. ψ Significantly different from pretreatment value, *p* < 0.05.

Since anilines and nitrobenzenes can be metabolized to each other, further studies were designed to explore the role of renal biotransformation and free radicals in cytotoxicity following exposure to 1.0 mM 3,5-DCNB for 60 min. To determine the potential role of free radicals in 3,5-DCNB cytotoxicity, IKC were pretreated with an antioxidant. Pretreatment with all antioxidants (α-tocopherol, glutathione, ascorbate, and N-acetyl-L-cysteine; Figure 3) significantly attenuated 3,5-DCNB-induced cytotoxicity.

**Figure 4:**
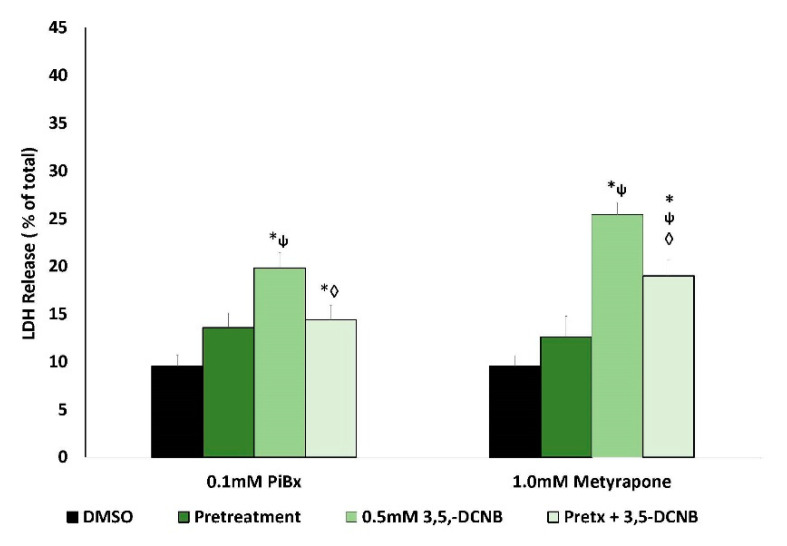
Effects of nonselective cytochrome P450 inhibitor pretreatment (Pretx) on 3,5-DCNB induced cytotoxicity in isolated kidney cells obtained from male Fischer 344 rats after 60 min. Each bar represents the mean ± S.E.M. for N = 4–5 separate isolation experiments. * Significantly different from DMSO control, *p* < 0.05. ◊ Significantly different from the 1.0 mM 3,5-DCNB value, *p* < 0.05. ψ Significantly different from pretreatment value, *p* < 0.05.

**Figure 5:**
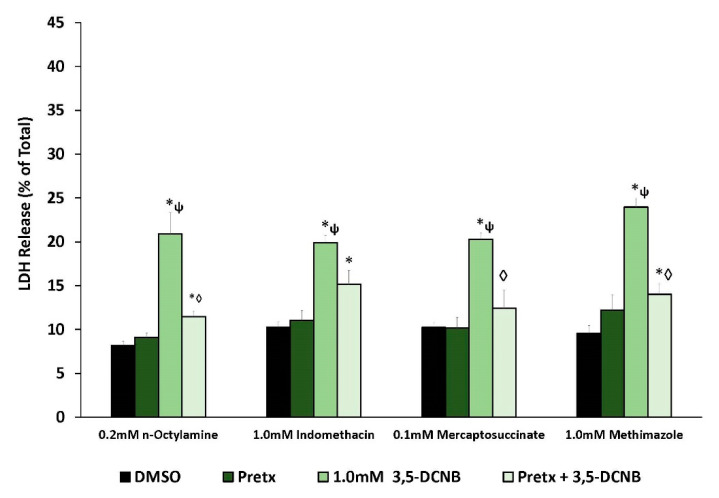
Effects of FMO, cyclooxygenase, and peroxidase inhibitor pretreatment (Pretx) on 3,5-DCNB cytotoxicity in isolated kidney cells obtained from male Fischer 344 rats after 60 min. Each bar represents the mean ± S.E.M. for N = 4–5 separate isolation experiments. * Significantly different from DMSO control, *p* < 0.05. ◊ Significantly different from the 1.0 mM 3,5-DCNB value, *p* < 0.05. ψ Significantly different from pretreatment value, *p* < 0.05.

To explore the role of renal biotransformation in 3,5-DCNB toxicity in IKC, pretreatment with inhibitors of cytochromes P450 (CYPs), flavin-containing monooxygenases (FMOs), and peroxidases was utilized. The use of CYP inhibitors (Figure 4) showed that two general CYP inhibitors (piperonyl butoxide [PiBx]; metyrapone) significantly attenuated 3,5-DCNB cytotoxicity. Pretreatment with FMO inhibitors (n-octylamine; methimazole), and the peroxidase inhibitor mercaptosuccinate, significantly attenuated 3,5-DCBN cytotoxicity (Figure 5). Indomethacin, which inhibits the peroxidase activity of cyclooxygenase tended to reduce 3,5-DCNB-induced LDH release but did not reach a significant difference (Figure 5).

### 2.3. Antioxidants and 3,5-DCPHA Cytotoxicity

**Figure 6:**
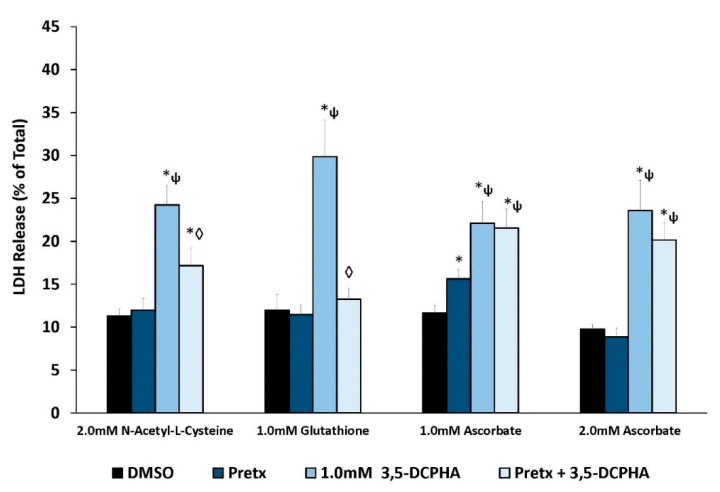
Effects of antioxidant pretreatment (Pretx) on 3,5-DCPHA induced cytotoxicity in isolated kidney cells obtained from male Fischer 344 rats after 60 min. Each bar represents the mean ± S.E.M. for N = 4–5 separate isolation experiments. * Significantly different from DMSO control, *p* < 0.05. ◊ Significantly different from the 0.5 mM 3,5-DCPHA value, *p* < 0.05. ψ Significantly different from pretreatment value, *p* < 0.05.

To begin to explore the role of free radicals in 3,5-DCPHA-induced cytotoxicity, IKC were pretreated with antioxidants (glutathione, ascorbate or N-acetyl-L-cysteine) prior to exposure to 3,5-DCPHA (0.5 mM). Results show that, in contrast to 3,5-DCNB, 3,5-DCPHA induced cytotoxicity was partially attenuated with N-acetyl-L-cysteine and fully protected by glutathione (Figure 6). Ascorbate (1.0 or 2.0 mM) was not effective at reducing 3,5-DCPHA cytotoxicity.

### 2.4. 3,5-DCA Metabolism by IKC

The previous comparative cytotoxicity studies were designed to explore the nephrotoxic potential of putative 3,5-DCA metabolites produced extra-renally. We also examined how IKC metabolized 3,5-DCA to see which metabolites were directly produced by the kidney cells. In these experiments, IKC were incubated with either a nontoxic concentration of 3,5-DCA (0.5 mM) or a cytotoxic concentration (1.0 mM) for 90 min and samples of media and cells analyzed via HPLC for 3,5-DCA and its metabolites. Retention times for 3,5-DCA and its metabolites, limits of detection, and extraction coefficients are shown in Table 1 for authentic standards of 3,5-DCA and each metabolite (4-A-2,6-DCP, 2-A-4,6-DCP, 3,5-DCPHA, 3,5-DCAA and 3,5-DCNB).

At both concentrations, the major compound in media and cells at the end of the incubation period was the parent compound 3,5-DCA. Less than 5% of 3,5-DCA was metabolized to 3,5-DCAA and 3,5-DCNB (Table 2). There was no evidence for the formation of unconjugated aminochlorophenol metabolites 2-A-4,6-DCP or 4-A-2,6-DCP. 3,5-DCPHA was not detected, which was not surprising given the highly reactive nature of this metabolite, but the finding that 3,5-DCNB was present in media and cells supports the conclusion that 3,5-DCPHA was formed and then further oxidized to 3,5-DCNB.

The possibility existed that the aminochlorophenols were formed but metabolized to sulfate or glucuronide conjugates. To determine if conjugates were formed, 3,5-DCA (1.0 mM) was incubated with IKC for 45 or 90 min. However, treatment of media or cells lysates with arylsulfatase or β-glucuronidase did not reveal any free 2-A-4,6-DCP or 4-A-2,6-DCP (data not shown). These results suggest that these two metabolites were not formed at detectable levels or were further oxidized to reactive metabolites (Figure 1). However, since essentially full recovery of 3,5-DCA equivalents occurred, it is unlikely that very much, if any, of these metabolites were formed by the kidney cells.

### 2.5. Effect of Diethyldithiocarbamic Acid (DEDTCA) on 3,5-DCA Metabolism

Previous studies had demonstrated that pretreatment of IKC with CYP2C inhibitors markedly reduced 3,5-DCA cytotoxicity [25]. One of the most effective inhibitors of 3,5-DCA toxicity was the CYP2C inhibitor DEDTCA. To examine how DEDTCA affected 3,5-DCA metabolism, IKC were pretreated with DEDTCA (0.1 mM) followed by 3,5-DCA (1.0 mM) for a 90 min incubation. Determination of 3,5-DCA and 3,5-DCA metabolite levels revealed that the total level of 3,5-DCA in media and cells was only slightly increased, while formation of 3,5-DCAA and 3,5-DCNB were reduced (Table 3). There was no 2-A-4,6-DCP, 4-A-2,6-DCP or 3,5-DCPHA detected, which agrees with earlier studies (Table 2).

## 3. Discussion

An important aspect of this study was determining the nephrotoxic potential of putative extra-renally produced metabolites of 3,5-DCA on renal target cells. These metabolites are predicted to be formed based on the metabolism of aniline and chloroanilines (Figure 1) [13,26,28,29]. We have previously calculated that a nephrotoxic dose of 3,5-DCA in a Fischer 344 rat would yield a maximum blood level of ~1.25 mM [25]. Thus, concentrations of metabolites below this concentration could be physiologically relevant. Results of the current study show that the metabolite arising from N-acetylation of 3,5-DCA, 3,5-DCAA, possessed a greatly reduced nephrotoxic potential relative to 3,5-DCA. This result is not surprising and is in agreement with earlier studies with monochloroanilines and their metabolites which show that N-acetylation results in chloroacetanilide metabolites with reduced nephrotoxic potential as compared to the parent compound [35,36]. Thus, N-acetylation appears to be a detoxification pathway for chloroaniline-induced nephrotoxicity, including 3,5-DCA. In addition, IKC are able to metabolize 3,5-DCA to 3,5-DCAA (Table 2). But given the weak nephrotoxic potential of 3,5-DCAA, it is unlikely that this metabolite contributes to 3,5-DCA induced nephrotoxicity in vitro.

In contrast to 3,5-DCAA, at least one metabolite resulting from phenyl ring oxidation of 3,5-DCA induces nephrotoxicity. Oxidation of 3,5-DCA at the 4-position, produces 4-A-2,6-DCP (Figure 1), which is a potent nephrotoxicant in vivo and in vitro [31,32,33]. In vivo, 4-A-2,6-DCP (0.38 mmol/kg, i.p.) induces marked nephrotoxicity in male Fischer 344 rats characterized by proteinuria, glucosuria, elevated blood urea nitrogen (BUN) concentration and kidney weight, and proximal tubular necrosis in the S_3_ segment [31,32]. In vitro, 4-A-2,6-DCP reduced uptake of p-aminohippurate (PAH) and tetraethylammonium (TEA) in rat renal cortical slices at concentrations of 5 µM and 50 µM, respectively [31] and increased LDH release at 50 µM or higher after 2 h exposure [33]. In IKC, concentrations of 250 µM or higher and a 90 min exposure are required for 4-A-2,6-DCP to increase LDH release. The higher concentrations needed to induce cytotoxicity in this model are most likely due to the presence of albumin in the incubation media for IKC but not in the Krebs–Ringer solution used in renal cortical slice incubation studies. In this study, 2-A-4,6-DCP, which results from oxidation at the 2-position of 3,5-DCA, did not increase LDH release at concentrations up to 1.5 mM and up to a 90 exposure, indicating that 4-A-2,6-DCP was more potent as a nephrotoxicant than 2-A-4,6-DCP. This finding is in agreement with comparisons of the nephrotoxic potentials of other 4-amino- and 2-aminophenols [37,38]. Thus, 4-A-2,6-DCP appears more likely than 2-A-4,6-DCP to contribute to 3,5-DCA-induced nephrotoxicity in vivo.

In the IKC 3,5-DCA metabolism studies, neither 2-A-4,6-DCA nor 4-A-2,6-DCA were detected as free metabolites or as glucuronide or sulfate conjugates (Table 2). While it is possible that small amounts of these aminodichlorophenols were produced, a complete recovery of the 3,5-DCA added to the IKC incubation was detected as parent compound plus other metabolites (Table 2). These results suggest that the kidney is not forming sufficient aminodichlorophenol metabolites to contribute to 3,5-DCA nephrotoxicity in vitro.

N-Oxidation has been shown to be an important biotransformation pathway of aniline and chloroaniline metabolism which can contribute to hematotoxicity, and potentially other toxicities, induced by these compounds [11,39,40,41]. N-Oxidation of an aniline produces the phenylhydroxylamine metabolite that is further oxidized to the nitrosobenzene metabolite [39] (Figure 1). The phenylhydroxylamine and nitrosobenzene metabolites can redox cycle to generate free radicals and oxidize ferrous iron in hemoglobin, and other ferrous iron containing proteins, to ferric iron. This cycling converts hemoglobin to methemoglobin in erythrocytes but can also affect the biological activity of other proteins and enzymes that require a ferrous iron molecule for biological activity. The nitrosobenzene metabolites are also reactive and can covalently bind to nucleophiles on macromolecules within a cell [42,43]. The nitroso group may be further oxidized to form nitrobenzene metabolites (Figure 1). Previous studies have demonstrated that 3,5-DCA can induce methemoglobinemia in vivo and that 3,5-DCPHA (Figure 1) is primarily responsible for this toxicity [40,44,45]. These findings suggest that 3,5-DCPHA is being produced extra-renally, most likely in the liver, and moving into the blood to induce methemoglobinemia as occurs with aniline [44,45]. These findings also suggest that the kidney is being exposed to 3,5-DCPHA produced at extra-renal locations.

The formation of 3,5-DCNB by IKC (Table 2), demonstrates that the kidney can form 3,5-DCPHA from 3,5-DCA. Thus, the kidney can be exposed to 3,5-DCPHA from both intra and extra-renal formation. 3,5-DCPHA proved to be the most toxic metabolite tested, and it is likely that 3,5-DCPHA is a major contributor to 3,5-DCA nephrotoxicity, directly to kidney cells and/or indirectly via hematotoxicity. It is also likely that multiple enzyme systems are capable of forming 3,5-DCPHA from 3,5-DCA, and these enzyme systems may also be forming other 3,5-DCA nephrotoxic metabolites [46,47]. N-Oxidation of aniline compounds has been shown to be catalyzed by CYPs, flavin monooxygenases (FMOs), peroxidases and prostaglandin synthetase [48,49,50,51]. Previous studies from our laboratory have shown that 3,5-DCA nephrotoxicity in IKC can be attenuated by inhibitors of these various enzyme families to varying degrees [25], suggesting that inhibition of one enzyme family will allow 3,5-DCA to continue to be bioactivated by other metabolizing enzyme systems. To determine if the inhibitors used by Racine et al. [25] were altering N-oxidation of 3,5-DCA, the ability of diethyldithiocarbamate (DEDTCA), an inhibitor of CYP2C, and to a lesser extent CYP2E1, to reduce DCNB formation was examined. DEDTCA was selected for study because it markedly reduced 3,5-DCA cytotoxicity in IKC [25]. The finding that DEDTCA reduced the formation of 3,5-DCNB in IKC supports the conclusion that DEDTCA attenuated 3,5-DCA nephrotoxicity, at least in part, by inhibiting N-oxidation of 3,5-DCA. Since the CYP2E1 inhibitor isoniazid had no effect on 3,5-DCA cytotoxicity, it appears that CYP2C enzymes are bioactivating 3,5-DCA to toxic metabolites via 3,5-DCPHA formation.

Cytotoxicity can be produced in vitro in IKC by 3,5-DCPHA via two potential mechanisms; redox cycling to produce free radicals (e.g., superoxide anion radical) and oxidative stress or production of an alkylating metabolite(s). Racine et al. [25] found that 3,5-DCA-induced cytotoxicity in IKC was not due to oxidative stress. Reduced and total glutathione levels were both reduced by 3,5-DCA exposure suggesting formation of a reactive metabolite. In the current study, 3,5-DCPHA cytotoxicity was reduced by nucleophilic antioxidants (glutathione and N-acetyl-L-cysteine), but not by the non-nucleophilic antioxidant ascorbate (Figure 6). These results support one or more reactive, alkylating metabolites of 3,5-DCPHA as the cytotoxic species.

Chlorinated nitrobenzenes are nephrotoxicants in vivo and in vitro [34,52,53]. Rickert and Held [3] found that hepatic microsomes from male Fischer 344 rats metabolized 3-chloronitrobenzene by reduction of the nitro group to form 3-chloroaniline, indicating that metabolism between 3,5-DCA and 3,5-DCNB is reversible. Thus, reduction of 3,5-DCNB will produce 3,5-dichloronitrosobenzene and 3,5-DCPHA just as oxidation of 3,5-DCA produces these two metabolites. However, the potential contribution of 3,5-DCNB to 3,5-DCA nephrotoxicity was unclear. If 3,5-DCNB was a nephrotoxic metabolite, it was also unclear whether biotransformation was required to produce the toxic chemical species. The current results support 3,5-DCNB as a toxic 3,5-DCA metabolite, potentially contributing to 3,5-DCA nephrotoxicity following biotransformation.

The current study demonstrates that 3,5-DCNB is a more potent nephrotoxicant than 3,5-DCA in IKC [25]. In addition, the ability of antioxidants and inhibitors of oxidative biotransformation to attenuate 3,5-DCNB cytotoxicity mirrors the effects of these pretreatments in reducing 3,5-DCA cytotoxicity in IKC [25]. These results suggest that any 3,5-DCNB formed during 3,5-DCA biotransformation has the potential to contribute to 3,5-DCA nephrotoxicity via metabolism of 3,5-DCNB being converted back to 3,5-dichloronitrsobenzene and 3,5-DCPHA. However, the weak activity of the CYP inhibitors on 3,5-DCNB cytotoxicity suggests that other enzyme systems (e.g., non-CYP nitroreductases) may be responsible for, or contribute to, the reduction of the nitro group in this model system. In addition, the ability of the antioxidants to markedly attenuate 3,5-DCNB cytotoxicity supports the role of nitro group-derived free radicals and/or reactive oxygen/nitrogen species in the nephrotoxic mechanism for this compound. Given the marked attenuation of 3,5-DCNB nephrotoxicity by some of the inhibitors of oxidative metabolism (e.g., n-octylamine and mercaptosuccinate), it is unlikely that 3,5-DCNB is directly toxic to kidney cells but produces its cytotoxicity via metabolites. In addition, the in vitro 3,5-DCA metabolism studies suggest that the renal cells may not make sufficient 3,5-DCNB to be responsible for 3,5-DCA cytotoxicity in vitro. It is possible that extrarenal metabolism of 3,5-DCA produces sufficient 3,5-DCNB to contribute to 3,5-DCA-induced nephrotoxicity in vivo, but the overall contribution of 3,5-DCNB to 3,5-DCA nephrotoxicity will require further study.

In summary, the results of these studies suggest that extra-renally generated aminodichlorophenol metabolites and metabolites arising from N-oxidation have the potential to contribute to 3,5-DCA nephrotoxicity in vivo, while N-acetylation of 3,5-DCA is a detoxification mechanism. In vitro, N-oxidation of 3,5-DCA is the major mechanism of bioactivation of 3,5-DCA to cytotoxic metabolites, and toxicity is most likely mediated by 3,5-dichloronitrsobenzene and/or 3,5-DCPHA. In addition, 3,5-DCNB-induced nephrotoxicity is due to the formation of metabolites, and nephrotoxicity of 3,5-DCNB is also most likely is mediated by 3,5-dichloronitrsobenzene and/or 3,5-DCPHA.

## 4. Materials and Methods

### 4.1. Animals

Male Fischer 344 rats (200–250 g) were obtained from Hilltop Lab Animals, Inc. (Scottsdale, PA, USA). Animals were housed two rats per cage with food and water available ad libitum, with temperature (21–23 °C), humidity (40–55%), and light (12 h on/12 h off) controlled. Prior to use, all animals were allowed at least one week to acclimate in American Association for Accreditation of Laboratory Animal Care (AAALAC) accredited animal facilities. All animal use was approved by the Marshall University Institutional Animal Care and Use Committee (Protocols 447 (5 August 2010) and 531 (1 February 2013)), and animal use experiments were conducted in accordance with the Guide for the Care and Use of Laboratory Animals, adopted by the National Institute of Health.

### 4.2. Chemicals

All chemicals were purchased at the highest purity available from either Fischer Scientific (Pittsburgh, PA, USA) or Sigma Aldrich (St. Louis, MO, USA), except for 3,5-dichlorophenylhydroxylamine (3,5-DCPHA), 3,5-dichloroacetanilide (3,5-DCAA), and 2-amino-4,6-dichlorophenol (2-A-4,6-DCP), which were synthesized in our laboratory. 3,5-DCPHA was synthesized by reduction of 3,5-DCNB using hydrazine-palladium on carbon via the previously described method of Rondestvedt and Johnson [54] and purified as described by Valentovic et al. [55]. Synthesis and purification of 3,5-DCAA was accomplished using the method of Newell et al. [56] by reacting equal amounts of 3,5-dichloroaniline and acetic anhydride in the presence of sodium acetate. To prepare 2-A-4,6-DCP, 2,4-dichlorophenol was nitrated using the method of McMillan et al. [29,57] followed by reduction of the nitro group to an amino group with sodium hydrosulfite in 10% aqueous sodium hydroxide according to Newell et al. [56].

### 4.3. Preparation and Treatment of Isolated Kidney Cells

Isolated kidney cells (IKC) were obtained from untreated male Fischer 344 rats. Male Fischer 344 rats were selected as the model to obtain isolated kidney cells as the majority of our previous toxicity data with 3,5-DCA has been obtained in this model. Rats were anesthetized with pentobarbital (75 mg/kg, i.p.), and IKC prepared via the method of Jones et al. [58] using collagenase perfusion as described by Racine et al. [25]. Kidney cells isolated by this technique are enriched in renal cortical cells based on biochemical characterization and are frequently used in renal toxicology assessments [59,60,61,62,63]. Initial cell viability was determined to be ~85–90% by lactate dehydrogenase (LDH) release and trypan blue (2% *w/v*) exclusion. Prior to incubation, IKC were counted and resuspended in Krebs–Henseleit (pH 7.37; 25 mM Hepes; 2% *w/v* bovine serum albumin) buffer at a concentration of ~4.2 million cells/mL. IKC (3 mL) were pre-incubated in 25 mL polycarbonate Erlenmeyer flasks for 5 min at 37 °C under an atmosphere of 95% oxygen/5% carbon dioxide. Following pre-incubation, cells were exposed to various concentrations of 3,5-DCNB (0.5–1.5 mM), 3,5-DCAA (0.5–1.5 mM), 3,5-dichlorophenylhydroxylamine (3,5-DCPHA; 0.25–1.0 mM), 2-A-4,6-DCP (0.5–1.5 mM), or vehicle (30 μL DMSO) for up to 90 min. At the conclusion of the allotted incubation period, samples (0.5 mL) were taken for the LDH release assay, as previously described [20,25].

To determine the role of renal biotransformation in 3,5-DCNB cytotoxicity and free radicals in 3,5-DCPHA nephrotoxicity, IKC were pretreated with either an antioxidant or an inhibitor of biotransformation enzyme systems (Table 4) or vehicle (30 µL DMSO) prior to exposure to 1.0 mM 3,5-DCNB (60 min) or 0.5 mM 3,5-DCPHA (60 min). All concentrations and pretreatment times of the antioxidants and renal enzyme inhibitors were based on previously published studies [20,25].

### 4.4. IKC Metabolism of 3,5-DCA

#### 4.4.1. IKC Incubations and Metabolite Isolation

IKC (~4.2 million cells/mL; 3 mL) were incubated with 3,5,-DCA (0.5 or 1.0 mM) in Krebs–Henseleit (KH) buffer (pH 7.4) for 90 min in a shaking water bath under a 95% oxygen/5% carbon dioxide atmosphere as described above. The 0.5 mM concentration of 3,5-DCA is a non-cytotoxic concentration at 90 min, while the 1.0 mM concentration induces cytotoxicity as evidenced by increased LDH release. At the end of the incubation period, 1.0 mL samples were collected, and media and cells separated by centrifugation. The pelleted cells were washed with KH buffer (no bovine serum albumin), and the rinse was added to the media. Cells were lysed via sonication and protein was precipitated by methanol (1.0 mL) addition to all samples. Samples were centrifuged (3000 g; 10 min, 4 °C) and the supernatant filtered through a 0.45 µ syringe filter and stored at −20 °C until analyzed by HPLC as described below.

#### 4.4.2. Determination of Sulfate and Glucuronide Conjugates

To determine if sulfate or glucuronide conjugates were formed, in some experiments, media and cell supernatant fractions obtained as described above were treated with β-glucuronidase (6500 units/mL, final concentration) or arylsulfatase (200 units/mL, final concentration) + saccaharolactone (20 mM, final concentration) for 18 h (37 °C) in 0.1 M acetate buffer (pH 5.0) to hydrolyze any glucuronide or sulfate conjugates, respectively, that were formed [26]. Hydrolysis was stopped by the addition of cold methanol (2.4 mL). The fractions were then filtered and stored until analyzed as described above.

#### 4.4.3. Inhibition of CYP2C Metabolism by DEDCTA

In a separate experiment, cells were pretreated with diethyldithiocarbamic acid (DEDTCA, 0.1 mM, 30 min pretreatment), a CYP2C inhibitor, before 3,5-DCA addition to determine which metabolites were decreased with DEDTCA pretreatment. Following the 90 min incubation period, cells and media were treated and stored as described above for the determination of 3,5-DCA concentrations and the concentrations of 3,5-DCA metabolites formed.

#### 4.4.4. HPLC Determination of Metabolites

Samples (75 µL) of media and lysed IKC were analyzed on a Waters Alliance e2695 HPLC system (Waters Co., Milford, MA, USA), utilizing a Waters 2489 variable UV/Vis detector at 254 nM. Chromatograms were collected and integrated using Empower 3 software (Waters Co., Milford, MA, USA). A Waters X Select HSS T3 C18 column (3.5 µm; 4.6 × 150 mm^2^) fitted with a Waters XSelect HSS T3 VanGuard Cartridge (3.5 µm; 3.9 × 5 mm^2^) was used for separation of compounds. The mobile phase consisted of 50:50 methanol:water with a flow rate of 0.75 mL/min. Standard curves, limits of detection, and extraction coefficients were determined for 3,5-DCA and putative metabolites. Extraction coefficients were determined by comparing the integration of a 1.0 mM 3,5-DCA or a metabolite sample that had undergone sample processing (i.e., the addition of methanol and centrifugation) to that of a unprocessed 1.0 mM 3,5-DCA or metabolite sample.

### 4.5. Statistics

Data are presented as mean ± S.E.M. with an N of 4–5 separate animal experiments, with the exception of *N* = 3 in the nontoxic 0.5 mM 3,5-DCA metabolism experiment. Data between treatments were analyzed by one-way analysis of variance (ANOVA) followed by a Tukey Test. Significance was determined at *p* < 0.05. A power analysis was performed with G * Power software version 3.1.9.7 for F tests, ANOVA (repeated measures, within factors) with effect size f = 0.85, α error probability = 0.05, power (1-β error probability) = 0.8 and actual power = 0.832, which determined a sample size of n = 4 was sufficient with a 95% confidence interval.

## Figures and Tables

**Table 1:** Retention times, limits of detection and extraction efficiency of 3,5-DCA and its metabolites.

Compound	Retention Time (Min)	Limit of Detection (µg)	Extraction Efficiency (%)
4-A-2,6-DCP	3.8	0.018	75.8
2-A-4,6-DCP	13.8	0.018	97.8
3,5-DCPHA	24.6	17.8	0.0 *
3,5-DCA	29.4	0.002	89.5
3,5-DCAA	42.6	0.002	96.1
3,5-DCNB	57.7	0.002	79.7

* 3,5-DCPHA rapidly degraded and the pure compound was not extractable.

**Table 2:** Metabolism of 3,5-DCA in isolated kidney cells (IKC) *.

Compound	0.5 mM		1.0 mM	
	Percentage of Dose (%)			
	Media	Cells	Media	Cells
4-A-2,6-DCP	ND	ND	ND	ND
2-A-4,6-DCP	ND	ND	ND	ND
3,5-DCPHA	ND	ND	ND	ND
3,5-DCA	89.31 ± 6.32	6.82 ± 3.33	87.13 ± 4.56	13.19 ± 2.12
3,5-DCAA	1.86 ± 0.42	0.07 ^	0.56 ± 0.07	ND
3,5-DCNB	2.80 ^	0.41 ^	1.18 ± 0.57 ^#^	0.34 ± 0.19 ^#^
Total	93.97 ± 5.52	7.30 ± 3.32	88.83 ± 5.14	13.53 ± 5.14

* IKC were incubated with 3,5-DCA at 0.5 mM (N = 3; nontoxic) or 1.0 mM (N = 4; cytotoxic) for 90 min. Cells and media were separated and the amount of 3,5-DCA and its metabolites in each fraction was determined by HPLC. Data are presented as mean ± SE. ^ Detected in only one sample. ^#^ Detected in three samples. ND = not detected.

**Table 3:** Effect of DEDTCA on 3,5-DCA metabolism in IKC *.

Compound	−DEDTCA		+DEDTCA	
	Percent of Dose (%)			
	Media	Cells	Media	Cells
3,5-DCA	89.43 ± 5.66	16.01 ± 2.02	91.14 ± 3.04	14.67 ± 1.78
3,5-DCAA	0.50 ± 0.03	0.02 ^	0.30 ± 0.05	ND
3,5-DCNB	0.31 ± 0.19 ^#^	0.13 ^	0.09 ^	0.06 ^

* IKC were pretreated with DEDTCA (0.1 mM) for 30 min followed by 3,5-DCA (1.0 mM) and the incubation continued for 90 min. Values are means ± SE for N = 4 separate experiments. ^ Only found in one sample. ^#^ Only found in two samples. ND = not detected.

**Table 4:** Pretreatments and Conditions *.

Pretreatment	Concentration	Pretreatment Time	Mechanism of Action
	(mM)	(Min)	or Enzyme Inhibited
α-Tocopherol	1	5	Antioxidant
Ascorbate	1.0 or 2.0	5	Antioxidant
Glutathione	1	30	Antioxidant
N-Acetyl-L-Cysteine	2	30	Antioxidant
Piperonyl Butoxide	1	15	CYP General
Metyrapone	1	5	CYP General
n-Octylamine	2	5	FMO
Methimazole	1	30	FMO
Indomethacin	1	15	Cyclooxygenaase
Mercaptosuccinate	0.1	15	Peroxidase
DEDTCA	0.1	30	CYP2C > CYP2E

* Pretreatments were added to IKC at the concentration and time indicated prior to addition of 3,5-DCA or a 3,5-DCA metabolite [20,25].

## Data Availability

The data presented in this study are available on request from the corresponding author.

## Abbreviations

2-A-4,6-DCP	2-Amino-4,6-dichlorophenol
3,5-DCA	3,5-Dichloroaniline
3,5-DCAA	3,5-Dichloroacetanilide
3,5-DCNB	3,5-Dichloronitrobenzene
3,5-DCPHA	3,5-Dichlorophenylhydroxylamine
4-A-2,6-DCP	4-Amino-2,6-dichlorophenol
ASC	Ascorbate
CYP	Cytochrome P450
DEDTCA	Diethyldithiocarbamate
DMSO	Dimethyl sulfoxide
FMO	Flavin-containing monooxygenase
GSH	Glutathione
IKC	Isolated kidney cells
LDH	Lactate dehydrogenase
NAC	N-Acetyl-L-cysteine
PiBx	Piperonyl butoxide
